# COVID-19 mitigation behaviors among English-Speaking Hmong Americans

**DOI:** 10.1186/s12889-023-15354-y

**Published:** 2023-03-14

**Authors:** Kao Kang Kue Vang, Sheryl Catz, Christiana Drake, Dian Baker, Lorena Garcia

**Affiliations:** 1 Betty Irene Moore School of Nursing, University of California Davis, 2570 48th Street, 95817 Sacramento, CA United States of America; 2 Department of Statistics, University of California Davis, Sacramento, CA United States of America; 3 School of Nursing, California State University, Sacramento, CA United States of America; 4 Department of Public Health Sciences-Division of Epidemiology, University of California Davis, Sacramento, CA United States of America

**Keywords:** Hmong Americans, Masking, Social Distancing, Group gatherings, COVID-19 vaccination

## Abstract

**Background:**

COVID-19 mitigation strategies such as masking, social distancing, avoiding group gatherings, and vaccination uptake are crucial interventions to preventing the spread of COVID-19. At present, COVID-19 data are aggregated and fail to identify subgroup variation in Asian American communities such as Hmong Americans. To understand the acceptance, adoption, and adherence to COVID-19 mitigation behaviors, an investigation of Hmong Americans’ contextual and personal characteristics was conducted.

**Methods:**

This study aims to describe COVID-19 mitigation behaviors among Hmong Americans and the contextual and personal characteristics that influence these behaviors. A cross-sectional online survey was conducted from April 8 till June 1, 2021, with Hmong Americans aged 18 and over. Descriptive statistics were used to summarize the overall characteristics and COVID-19 related behaviors of Hmong Americans. Chi-square and Fisher’s Exact Test were computed to describe COVID-19 mitigation behaviors by gender and generational status (a marker of acculturation).

**Results:**

The sample included 507 participants who completed the survey. A majority of the Hmong American participants in our study reported masking (449/505, 88.9%), social distancing (270/496, 55.3%), avoiding group gatherings (345/505, 68.3%), avoiding public spaces (366/506, 72.3%), and obtaining the COVID-19 vaccination (350/506, 69.2%) to stay safe from COVID-19. Women were more likely to socially distance (*P* = .005), and avoid family (*P* = .005), and social gatherings (*P* = .009) compared to men. Social influence patterns related to mitigation behaviors varied by sex. Men were more likely compared to women to be influenced by Hmong community leaders to participate in family and group gatherings (*P* = .026), masking (*P* = .029), social distancing (*P* = .022), and vaccination uptake (*P* = .037), whereas healthcare providers and government officials were social influencers for social distancing and masking for women. Patterns of social distancing and group gatherings were also influenced by generational status.

**Conclusion:**

Contextual and personal characteristics influence COVID-19 mitigation behaviors among English speaking Hmong Americans. These findings have implications for identifying and implementing culturally appropriate health messages, future public health interventions, policy development, and ongoing research with this population.

## Background

During the early onset of the pandemic, COVID-19 related deaths accelerated in ethnic minorities in the United States [1]. In a 2020 report of COVID-19 deaths in Minnesota, 49% of COVID-19 related deaths in the Asian American community were from Hmong Americans [2]. The lack of disaggregated race and ethnicity data including subgroups of Asian Americans prevents a full understanding of those affected by COVID-19. The paucity of studies, data, and reports specific to Hmong Americans during the COVID-19 pandemic limited understanding of their adherence to recommended mitigation practices. There are unique aspects of Hmong American collectivist culture and social influences for men and women of different generations that may impact the degree to which community members accept and adopt preventative health practices recommended during the COVID-19 era.

The Hmong are refugees who fled to the United States (U.S.) after the Vietnam War and for decades have experienced poverty, educational inequity, and health disparities [3, 4]. Hmong Americans immigrated to the United States in various waves. The first wave of Hmong Americans occurred in the 1970s, the largest wave in 1980s, followed by a smaller wave in 1990s, and lastly, the last wave in early 2000 [5]. With the differences in arrival to the United States, this impacts how Hmong Americans acclimated and acculturated to the U.S. mainstream as a whole group, resulting in the inability to achieve socioeconomic success [6, 7]. High morbidity and mortality seen in Hmong Americans are likely because of the underutilization and rejection of formal healthcare services, low use of preventative care, delay in seeking critical health services, and low health literacy [8–11]. Health behaviors in Hmong Americans are influenced by sociocultural beliefs and practices that include a complex infrastructure of cultural influences on healing practices, use of traditional medicine, religious beliefs, disease perception, social organization, and family roles [4, 8, 12, 13].

Hmong Americans’ conceptualization of medical conditions often don’t include preventive approaches or interventions due to their health beliefs. Hmong traditional views on health and illness, include natural and supernatural causes. Illnesses that are perceived to be caused by spiritual or supernatural causes will often result in the refusal to use Western healthcare services unless all other traditional practices have become futile [13]. Although many Hmong Americans engage in shamanistic rituals or traditional folk remedies to help maintain and restore health, not all Hmong Americans take part in such activities. Therefore, mitigation behaviors are particularly important to understand in Hmong Americans when historically issues have been seen with adherence to preventative interventions such as diet and lifestyle modification, medication compliance, cancer screening engagement, and vaccination uptake [8–11].

The Hmong American cultural system is complex, largely because it includes a patriarchal, patrilineal, clan-based, collectivist culture with gender roles utilizing a hierarchical approach to decision making. Despite living within an individualistic culture, it is not well understood if gender roles, clan, and male family influence impact preventative health behaviors. Traditionally, Hmong clan leaders or male heads of the household make life and health decisions for other individuals in their family by utilizing collectivist principles. A collectivist culture endorses that the group has priority over other individuals, therefore decisions are made within the group and problem solving is often done centrally [14, 15]. When individuals come from collectivist cultures, they are more tolerant of crowded living situations and maybe expected to participate in group rituals and practices [16]. In the context of COVID-19, this could make social distancing and self-isolation for multigeneration households and those with underlying health conditions at higher risk for COVID-19 [17]. While mitigation guidelines such as masking, social distancing, avoiding group gatherings, and vaccination uptake require the acceptance, adoption, and adherence of the individual; community and social context may be important drivers for individual health behaviors. Therefore, factors such as health beliefs and practices, cultural norms and expectations, family social structures, misinformation, trust, and influences from social constructs such as clan members, community leaders, and family members, need to be addressed to understand mitigation intervention effectiveness in Hmong Americans.

The lack of COVID-19 data on Hmong Americans further exacerbates limited understanding on COVID-19 related disparities, thereby restricting COVID-19 response policies and interventions aimed at this community. While resources and funding allocation are based on race/ethnicity data, missing or unavailable data prevent proper resource allocation [18]. Therefore, to design specific and tailored public health and healthcare messaging about COVID-19, more research is required to explain the barriers and facilitators for COVID-19 understanding and prevention among Hmong Americans. To our knowledge, there has been no survey on COVID-19 mitigation behaviors in Hmong Americans. Given that Hmong Americans as a group have a history of not fully engaging in community preventative interventions, and the importance to doing so during the COVID-19 pandemic, we sought to understand the extent to which Hmong Americans of different genders and generation status were able to take preventative action during the COVID-19 era. In view of this, the present study was designed to describe cross-sectional COVID-19 mitigation behaviors in Hmong Americans, as well as mitigation-related information, personal motivation, social motivation, and behavioral skills.

## Methods

### Theoretical framework

The Information-Motivation-Behavioral Skills (IMB) model was originally used to understand HIV-related risk behaviors [19] and has been adapted to articulate other preventive health behaviors and for other target populations. We applied the model to COVID-19, positing that adherence to masking, social distancing, avoidances of group gatherings, and vaccination uptake is a function of the individual’s knowledge of COVID-19 mitigation related information, motivation to carry out the prevention, and behavioral skills in conducting specific preventative behaviors [19, 20]. At the time of data collection there were no theory-based models specific to COVID-19 mitigation behaviors. A behavioral framework such as the Information Motivation Behavior Skills Model offered a reasonable conceptual framework for assessing mitigation behaviors, knowledge, and attitudes for the purpose of guiding public health interventions or education targeting a Hmong American population due to its ability to measure explicit relationships among constructs that are determinants of health behaviors including distinction between social and personal motivation that may be important for a collectivist culture within an individualist culture.

Currently little is known about how specific public health messaging design for the Hmong American community may be optimized. Therefore, theoretical evidence is needed to guide the development of specific culturally sensitive mitigation interventions targeting masking, social distancing, avoiding group gatherings, and vaccination uptake among Hmong Americans.

**Fig. 1:**
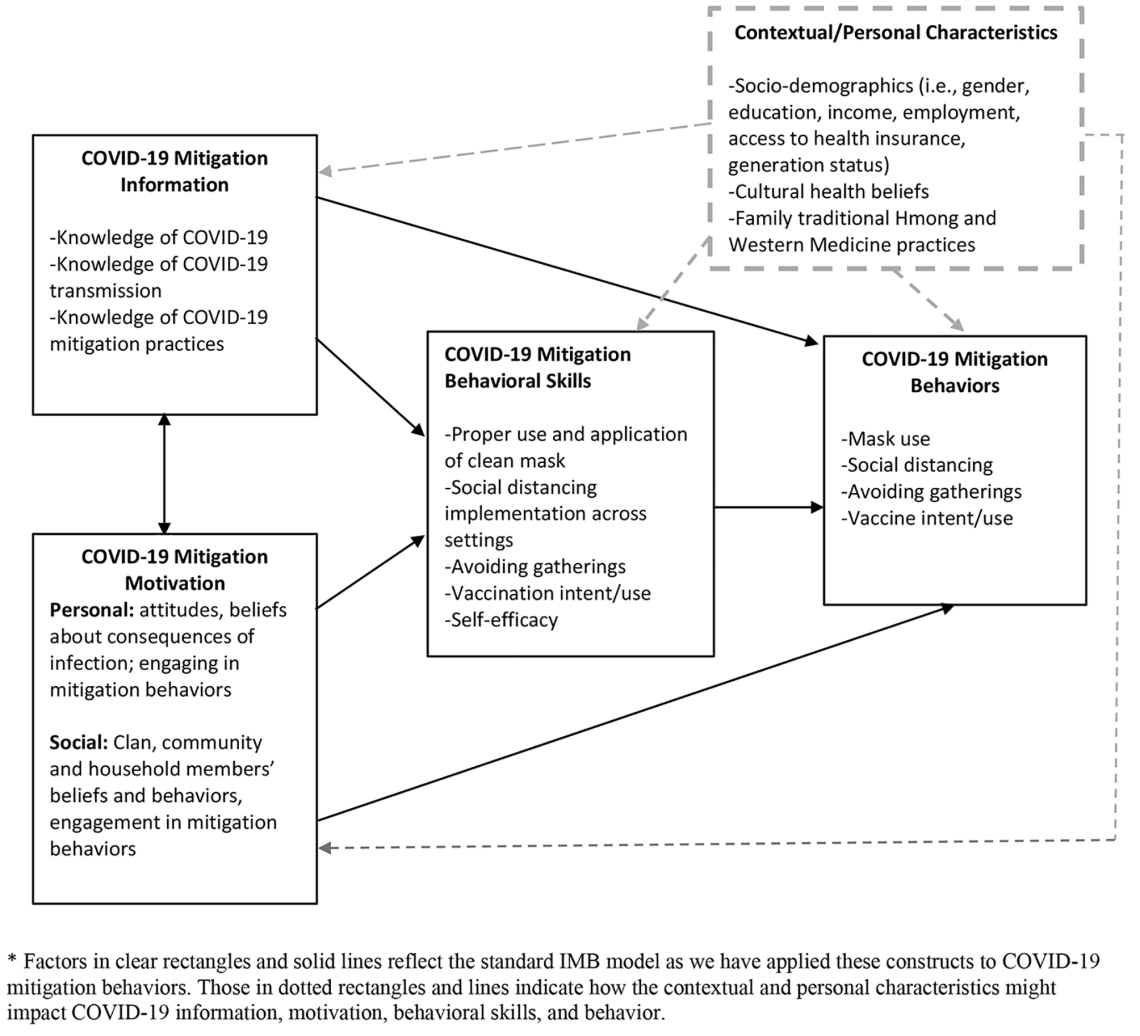
Expanded Information Motivational Behavioral Skills Model for COVID-19 Mitigation Behaviors in Hmong Americans

The version of the model that was used for this study included an expanded IMB model to include contextual and personal characteristics with the following constructs: (1) health behavior information, (2) health behavior motivation, (3) health behavioral skills, (4) health behaviors, and (5) contextual/personal characteristics (Fig. 1.)

### Study design and recruitment

To evaluate the adherence, adoption, acceptance of COVID-19 mitigation behaviors in Hmong Americans, a cross-sectional web survey was conducted from April 8 till June 1, 2021.

Recruitment methodology included digital advertisement, recruitment partners, word of mouth, social networks, and offline approaches. Community leaders, activists, and non-profit organizations from large Hmong American communities such as in California, Minnesota, Wisconsin, North Carolina, as well as other communities with strong Hmong organizations in Georgia and Oregon were contacted and asked to complete the survey and serve as a recruitment partner by encouraging their communities to complete the survey. Recruitment partners were asked to distribute information about the survey to the Hmong community. Information about the study, and digital recruitment materials such as digital fliers and a prerecorded video from the researcher were used to promote the study. To mitigate the mistrust that Hmong Americans often have of research [21], study invitation materials and a video clarified that the lead researcher was also Hmong American. The video campaign was 1 min and 47 s long in English. The video included information about the researcher, why the study is being conducted, who is eligible to participate in the study, researcher information and contact, and IRB approval statement from UC Davis Ethics Committee. Digital flyers included detailed information about the study, information about the researchers conducting the study, and the direct link and QR code to the survey. Individuals interested in the study were directed to a digital survey link. This was a voluntary anonymous survey, with no monetary incentives. The survey was conducted using a self-administered, anonymous questionnaire consisting of four sections: (1) sociodemographic, (2) COVID-19 knowledge and mitigation interventions, (3) personal and social motivation to adopt mitigation behaviors, and (4) mitigation behavioral skills. Survey responses were collected and stored in Qualtrics XM® (Provo, Utah) software.

### Survey development

Our two-step approach to developing a culturally appropriate survey assessing Hmong Americans was, first to identify an item pool based on existing tools used to measure COVID-19 preventative information, motivation, and mitigation behaviors skills, and second, cognitive interviewing. Cognitive interviewing using a think-aloud methodology with 5 Hmong American survey respondents informed our final decision on response anchors to avoid complex rating scales when possible and to use Yes/No options instead of True/False statements, because True/False statements lacked clarity for this population. The survey included range checks to make sure there wasn’t something out of range and reminders to complete missing items.

Surveys used to develop the initial item pool included the International COVID-19 Awareness, and Response Evaluation Survey, Social Psychological Survey of COVID-19, USC Center for Economic and Social Research on Understanding America Study on Coronavirus tracking survey, behavioral insight studies related to COVID-19 from the World Health Organization European Region, Consumers and COVID-19: Survey Results on Mask-Wearing Behaviors and Beliefs, CDC COVID-19 Community Survey Question Bank, and the Knowledge, Attitudes, and Practices toward COVID-19 among Chinese questionnaire [22–28]. Vaccination intent or use questions were measured by items adapted from a battery of items used to validate COVID-19 vaccinations intention and use from the Kaiser Family Foundation (KFF) COVID-19 survey [29]. There were no COVID-19 surveys that were adapted and validated in the Hmong American population at the time of this study.

### Data Analysis

All data are stored in Qualtrics XM database provided by the University of California Davis and then imported into STATA (version 15.1) for statistical analysis. Categorical data was reported by one-way frequency and percentages. Descriptive statistics were used to summarize the overall characteristics of the sample. Chi-squared analyses were used for bivariate analyses between health behaviors with gender and generation status. Fisher’s Exact Test (FET) was used in analyses when the expected value in a cell fell below 5. FET were used for generational status by health behaviors. The majority of the respondents retained in the sample provided completed data. There were some missing items that were handled with pairwise deletion in analysis. Data that were dropped from the analysis included respondents who did not complete the survey beyond the screening criteria questions. Outliers were assessed and addressed if it appeared that the value was in error. A p-value of < 0.05 was considered to indicate a statistically significant difference.

## Results

A total of 609 participants accessed the link to the web survey. Of the 609 participants, 103 (17%) were either a non-responder or did not complete the survey beyond the screening criteria about age, U.S. residency status, and identification as Hmong American were dropped from the analysis. Only 507 (83%) participants completed the survey in its entirety and were used for the analysis. The characteristics of the samples are summarized in Table 1. Analysis of the sample revealed that 78% (376/482) were female, 20.8% (100/482) were male, and 1.2% (6/482) preferred not to say. Of the 507 participants, 38.7% (196/507) identified as first-generation Hmong American, 59% (299/507) as second generation, and 2.4% (12/507) as third generation. As expected, the sample shown in Table 1 was relatively young with 90.2% (435/482 ) being under 45 years old, 80.3% (387/482) have a college degree, 80.1% (386/482) were employed, 61.4% (281/481) have an annual household income over $70,000, and 93% (448/482) have health insurance. The great majority of respondents did not have any chronic health conditions while 5.6% (26/464) had lung disease/asthma, 12.2% (57/469) had heart disease/high blood pressure, 7.5% (35/466) had diabetes, 1.1% (5/464) had kidney disease, 1.1% (5/464) had cancer, and 27.5% (129/469) had obesity. Of the 506 participants, 87 (17%) respondents identified as being diagnosed or sick with COVID-19 (Table 1). Questions about household characteristics showed that 67.3% (296/440) participants had at least one child in their household, and 22.6% (96/425) participants had at least one older individual living with them.

**Table 1:** Sample Characteristics

Characteristics	n (%)
**Gender**	
Male	100 (20.8)
Female	376 (78)
Prefer not to say	6 (1.2)
**Age**	
18–24 years	48 (10)
25–34 years	205 (42.5)
35–44 years	182 (37.8)
45–54 years	41 (8.5)
55–64 years	5 (1)
65–74 years	1 (0.2)
**Generation Status**	
First generation	196 (38.7)
Second generation	299 (59)
Third generation	12 (2.4)
**Education Level**	
Less than High School education	4 (0.8)
High School graduate, diploma, or equivalent	63 (13.1)
Trade Degree	28 (5.8)
College Degree	387 (80.3)
**Employment Status**	
Employed	386 (80.1)
Unemployed	48 (10)
Student	46 (9.5)
Retired	2 (0.4)
**Annual Income**	
Less than $30,000	39 (8.1)
$30,000-$70,000	123 (25.6)
$71,000-$100,000	103 (21.4)
Over $100,000	178 (37)
Prefer not to say	38 (7.9)
**Healthcare Coverage/Insurance**	
Yes	448 (93)
No	34 (7.1)
**Types of Healthcare Insurance**	
Medicaid/Medicare	72 (15.2)
I get my health insurance through my work	338 (71.3)
I buy my own health insurance	22 (4.6)
Veterans Affair/Military Insurance	7 (1.5)
Other	35 (7.4)
**Chronic Health Conditions**	
Lung Disease/Asthma	
Yes	26 (5.6)
No	438 (94.4)
Heart Disease/High Blood Pressure	
Yes	57 (12.2)
No	412 (87.9)
Diabetes	
Yes	35 (7.5)
No	431 (92.5)
Kidney Disease	
Yes	5 (1.1)
No	459 (98.9)
Cancer	
Yes	5 (1.1)
No	459 (98.9)
Obesity	
Yes	129 (27.5)
No	340 (72.5)
**Diagnosed or sick with COVID-19**	
Yes	86 (17)
No	420 (83)
**Knew someone with COVID-19**	
Immediate family member	
Yes	273 (56.1)
No	214 (43.9)
Relative	
Yes	366 (75.2)
No	121 (24.9)
Friend	
Yes	315 (64.7)
No	172 (35.3)
Someone other than family, relative or friend	
Yes	97 (19.9)
No	390 (80.1)
**Ages in household**	
Children Under 18 years old	
None	144 (32.8)
One	77 (17.5)
Two	86 (19.6)
Three	61 (13.9)
Four	50 (11.4)
Five and More	22 (5)
18 to 64 years	
None	12 (2.5)
One	22 (4.7)
Two	181 (38.5)
Three	81 (17.2)
Four	69 (14.7)
Five and More	105 (22.3)
> 65 years old	
None	329 (77.4)
One	58 (13.7)
Two	31 (7.3)
Three	4 (0.9)
Four	0 (0)
Five and More	3 (0.7)

### COVID-19 information and sources of information

The participants in this study had a relatively high level of COVID-19 knowledge and knowledge of mitigation interventions such as masking, social distancing, avoiding group gatherings, and COVID-19 vaccines (Table 2). The top five common sources of information that participants used to seek COVID-19 information included website or online pages (397/506, 78.5%), social media (369/506, 72.9%), conversations with family and friends (315/506, 62.3%), government officials (308/506, 60.9%), and medical institutions (277/506, 54.7%) (Table 2). Majority of the participants knew the signs and symptoms of COVID-19. As shown in Tables 2 and 96.1% (486/506) participants reported that COVID-19 can spread through close contact with infected individuals, 98.6% (499/506) reported that the virus can spread without showing symptoms, and 97.4% (492/506) reported COVID-19 can get them sick. Analysis of sources of misinformation revealed that 50.6% (255/504) participants reported having found sources of misinformation.

**Table 2:** COVID-19 Sources of Information and Information

				Yes n (%)	No n (%)
**Sources of Information**					
Television				262 (51.8)	244 (48.2)
Conversations with Friends and Family				315 (62.3)	191 (37.8)
Website or Online Pages				397 (78.5)	109 (21.5)
Social Media				369 (72.9)	137 (27.1)
Radio Stations				85 (16.8)	421 (83.2)
Government Official				308 (60.9)	198 (39.1)
Medical Institution				277 (54.7)	229 (45.3)
Hmong Community Leaders				30 (5.9)	476 (94.1)
Found Sources of Misinformation				255 (50.6)	249 (49.4)
**Symptoms of COVID-19**					
Fevers and Chills				502 (99.6)	2 (0.4)
Cough				457 (90.7)	47 (9.3)
Shortness of Breath				496 (98.4)	8 (1.6)
Sore Throat				391 (77.6)	113 (22.4)
Runny Nose				336 (66.7)	168 (33.3)
Muscle Ache				473 (93.9)	31 (6.2)
Headache				430 (85.3)	74 (14.7)
Fatigue				457 (90.7)	47 (9.3)
Diarrhea				385 (76.4)	119 (23.6)
Loss of Taste and Smell				494 (98)	10 (2)
**Information About COVID-19**					
There are medications that can treat COVID-19				155 (30.8)	349 (69.2)
There are Hmong Medication that can treat COVID-19				40 (7.9)	466 (92.1)
There is a cure for COVID-19				19 (3.8)	487 (96.2)
The COVID-19 virus spreads through close contact with infected individuals				486 (96.1)	20 (3.9)
The COVID-19 virus can spread without showing symptoms				499 (98.6)	7 (1.4)
To prevent getting COVID-19, individuals should avoid going to crowded public spaces				492 (97.4)	13 (2.6)
COVID-19 can get me sick				492 (97.4)	14 (2.6)
You are more likely to catch COVID-19 from a stranger than a family member				55 (10.9)	451 (89.1)

### Trusted and untrusted sources of information

We assessed the level of trust for various sources of information. Sources of information included television, conversations with friends and family, website or online pages, social media, radio stations, government officials, medical institutions, and Hmong community leaders. Not shown in a table, the top five trusted sources of information were from medical institutions (458/497, 92.2%), government officials (401/497, 80.7%), websites or online pages (371/502, 73.9%), television (271/495, 54.7%), and conversations with friends and family (224/497, 44.7%). Social media (194/503, 38.6%), radio stations (185/489, 37.8%), and Hmong community leaders (78/488, 16%) were perceived by fewer participants to be trusted sources of information. Sources of information that were perceived to be not trusted showed that Hmong community leaders (410/488, 84%), radio stations (304/489, 62.2%), social media (309/503, 61.4%), conversations with friends and family (277/501, 55.3%), and television (224/495, 45.3%) were the top five untrusted sources of information. Websites or online pages (131/506, 26.1%), government officials (96/506, 19.3%), and medical institutions (39/506, 7.8%) had fewer participants perceive these sources as untrusted sources of information.

### Personal and social motivation

Assessment of personal motivation showed that the majority of participants reported masks (401/498, 80.5%), social distancing (388/505, 77.5%), avoiding group gatherings (431/505, 85.3%), and COVID-19 vaccines (378/495, 76.4%) are effective interventions against COVID-19 (Table 3). Most of the participants (424/505, 84%) endorsed that they are willing to mask the whole time when attending gatherings. When assessing if one is willing to social distance, 89.9% (454/505) reported that they will maintain 6 feet from people outside of their household. When leaving home, only 55.3% (279/505) of the participants endorsed they would stay 6 feet from people outside of their household all the time. Responses about willingness to avoid gatherings showed that 76.1% (385/506) of the participants will avoid family gatherings such as birthday parties, funerals, weddings, ceremonial, or cultural celebrations, while 83.6% (422/505) of the participants will avoid social gatherings. COVID-19 vaccination uptake assessment shows that 70% (354/506) were willing to get the vaccine as soon as possible, 19.8% (100/506) will wait to see how the vaccine is working for other people, 4.5% (29/506) will only get the vaccine if it is required, and 5.7% (29/506) will definitely not get the vaccine.

**Table 3:** Individual and Social Motivation and COVID-19 Mitigation Behavioral Skills and Behavior

	Yes	No
	n (%)	n (%)
**Masking**		
Mask do not spread COVID	135 (26.8)	368 (73.2)
Masks keep me safe	370 (73.1)	136 (26.9)
Wearing masks is too much trouble	31 (6.1)	474 (93.9)
I know how to wear a mask	465 (91.9)	41 (8.1)
Masks always overs my nose, mouth, and chin	496 (98)	10 (2)
I wear a new mask every day	303 (59.9)	203 (40.1)
Willing to mask the whole time at gatherings	424 (84)	81 (16)
Last 30 days worn a mask	500 (99)	5 (1)
Masking is effective prevention against COVID-19	401 (80.5)	117 (19.5)
Mask all the time when leaving home	449 (88.9)	56 (10.1)
My family thinks mask can keep them safe from COVID-19	428 (88.8)	54 (11.2)
Wearing a mask is too much trouble for my family	35 (7.3)	447 (92.7)
My family masks all the time when leaving home	413 (85.7)	69 (14.3)
My family worn a mask the last 30 days	466 (96.9)	15 (3.1)
**Social Distancing**		
I know how far 6 feet is form other people	485 (95.9)	21 (4.1)
I know how to keep people from walking or standing too close to me	433 (85.6)	73 (14.4)
Willing to stay 6 feet apart from people outside the household	454 (89.9)	51 (10.1)
Last 30 days stayed 6 feet from people outside the household	446 (88.3)	59 (11.7)
Staying 6 feet apart is effective prevention against COVID-19	388 (77.5)	74 (22.5)
Maintain 6 feet when leaving home all the time	270 (55.3)	226 (44.7)
My family maintained 6 feet all the time when leaving home	275 (57.1)	207 (42.9)
My family knows how far 6 feet is from other people	409 (85)	72 (15)
My family stayed 6 feet from people outside the household the last 30 days	418 (86.7)	64 (13.3)
**Group Gatherings**		
I know how to say “no” to group gatherings	462 (91.3)	44 (8.7)
Willing to avoid family gatherings	385 (76.1)	121 (23.9)
Willing to avoid social gatherings	422 (83.6)	83 (16.4)
Last 30 days avoided gatherings of 10 or more	345 (68.3)	160 (31.7)
Last 30 days avoid public spaces, gatherings, or crowds	366 (72.3)	140 (27.7)
Avoiding public space, group gatherings, and crowds is effective prevention against COVID-19	431 (85.3)	74 (14.7)
My family avoided gatherings of 10 or more the last 30 days	343 (71.5)	137 (28.5)
My family avoided public spaces, gatherings or crowds the last 30 days	351 (72.8)	131 (27.2)
**Vaccination**		
COVID vaccines are available for free	471 (93.1)	35 (6.9)
I trust authorities who say COVID vaccines are safe	312 (61.7)	194 (38.3)
Trusted leaders say everyone should get the COVID vaccine as soon as possible	340 (67.2)	166 (32.8)
I know where to get the vaccine	478 (94.5)	28 (5.5)
I know when it is my turn to get the vaccine	465 (91.9)	41 (8.1)
Vaccination is effective prevention against COVID-19	378 (76.4)	117 (23.6)
Willing to get the vaccine as soon as possible	354 (70)	152 (30)
Wait to see how the vaccine is working for other people	100 (19.8)	406 (80.2)
Only get the vaccine if it is required	23 (4.5)	483 (95.5)
Definitely not get the vaccine	29 (5.7)	477 (94.3)
Received the vaccine	350 (69.2)	156 (30.8)
Will mask after getting vaccine	485 (96.2)	19 (3.8)
Will social distance after getting vaccine	459 (91.4)	43 (8.6)
Will attend gatherings after getting vaccine	283 (56.2)	221 (43.8)
Some people in my family don’t trust authorities who say COVID-19 vaccines are safe	217 (45.1)	264 (54.9)
Some of my family members received the COVID-19 vaccine	423 (87.8)	59 (12.2)

Assessment of social motivation showed that 88.8% (428/482) of the participants reported their family felt masking can keep them safe from COVID-19; 86.7% (418/428) of participants reported their family stayed 6 feet from people who do not live with them in the last 30 days; 72.8% (351/482) reported their family avoided public spaces, gatherings, and crowds in the last 30 days; and 45.1% (217/481) of the participants reported some people in their family did not trust authorities who say COVID-19 vaccines are safe. Participants reported that their family members motivation to perform mitigation behaviors as the following: 85.7% (413/482) of participants reported their family masked all the time when leaving home;57% (275/482) reported their family maintained 6 feet from other people when leaving home, and 85% (409/481) knew how far 6 feet is from other people. Group gathering motivation in family showed that 71.5% (343/480) participants reported their family members avoided family gatherings with more than 10 people the last 30 days and 72.8% (351/482) avoided public spaces, gatherings, and crowds in the last 30 days. When assessing vaccine behaviors in family, 87.8% (423/482) of the participants reported someone in their family received the COVID-19 vaccine (Table 3).

### Mitigation behavioral skills

Mitigation behavioral skills showed that 93.9% (474/505) of the participants felt that wearing a mask was not too much trouble, 91.9% (465/506) reported they knew how to put on a mask so that it fits well, and 98% (496/506) endorsed that their mask always covered their nose, mouth, and chin. Social distancing behavioral skills showed that 95.9% (485/506) of the participants knew how far 6 feet is from other people, and 85.6% (433/506) knew how to keep people from being too close to them. Group gathering behavioral skills show that 91.3% (462/506) of the participants knew how to say “no” when invited to big gatherings. Survey responses about COVID-19 vaccine information and behavioral skills showed that 93.1% (471/506) of the participants knew vaccines are available for free, 94.5% (478/506) knew where to get the vaccine, and 91.9% (465/506) knew when it was their turn to get the vaccine (Table 3).

### Mitigation behavior

Mitigation behavior assessment showed that 88.9% (449/506) of participants reported to masked and 55.3% (270/506) social distanced all the time when leaving home, 72.3% (366/506) avoided public space, gatherings and crowds the last 30 days, and 69.1% (350/506) obtained the COVID-19 vaccine. Behaviors after being vaccinated showed that 96.2% (485/504) of the participants will intend to continue wearing a mask, 91.4% (459/502) will intend to stay 6 feet away from people outside their household, and 56.2% (283/504) will intend to attend gatherings with big groups of people (Table 3).

### Contextual and personal characteristics Associated with Mitigation Behaviors

#### Gender

There were few differences in masking behaviors reported by men and women when leaving home and masking at gatherings. There were differences in social distancing behaviors between Hmong American men and women. Hmong American women were more willing to stay 6 feet from people outside the household compared to Hmong American men (x^2^ = 8.02, *P* = .005). A greater proportion of Hmong American women than men reported they will avoid family (x^2^ = 8.02, *P* = .005) and social gatherings (x^2^ = 6.91, *P* = .009). There was no statistical difference between gender and vaccination intent or uptake behaviors (Table 4).

**Table 4:** COVID-19 Mitigation Behaviors by Gender

	Total N = 482				
	Total N	Male (n = 106)	Female (n = 376)	p-value	Fisher’s Exact
**Willing to Mask the whole time at gatherings**	**482**			0.255	
Yes		85 (80.2)	318 (83.8)		
No		21 (19.8)	57 (15.2)		
**Masking all the time when leaving home**	**482**			0.092	
Yes		89 (84)	337 (89.9)		
No		17 (16)	38 (10.1)		
**Last 30 days worn a mask**	**482**				0.074
Yes		103 (97.2)	373 (99.5)		
No		3 (2.8)	2 (0.5)		
**Willing to stay 6ft from people outside the household**	**482**			0.005*	
Yes		88 (83)	346 (90.3)		
No		18 (17)	29 (7.7)		
**Maintaining 6ft all the time when leaving home**	**482**			0.777	
Yes		60 (56.6)	207 (55.1)		
No		46 (43.4)	169 (44.9)		
**Last 30 days stay 6ft from people outside the household**	**482**			0.938	
Yes		93 (88.6)	332 (88.3)		
No		12 (11.4)	44 (11.7)		
**Willing to Avoid Family Gatherings**	**482**			0.005*	
Yes		71 (67)	301 (80.1)		
No		35 (33)	75 (19.9)		
**Willing to Avoid Social Gatherings**	**482**			0.009*	
Yes		80 (75.5)	323 (86.1)		
No		26 (24.5)	52 (13.9)		
**Last 30 days avoided gatherings of 10 or more**	**482**			0.369	
Yes		69 (65.1)	262 (68.7)		
No		37 (34.9)	114 (30.3)		
**Last 30 days avoided public spaces, gatherings or crowds**	**482**			0.853	
Yes		76 (71.7)	273 (72.6)		
No		30 (28.3)	103 (27.4)		
**Will vaccinate, when COVID-19 vaccine is available**	**482**			0.556	
Yes		77 (72.6)	262 (69.7)		
No		29 (27.4)	114 (30.3)		
**Received the COVID-19 vaccine**	**482**			0.489	
Yes		71 (67)	265 (70.5)		
No		35 (33)	111 (29.5)		
**After getting vaccine, likely to mask**	**482**				0.153
Yes		99 (93.4)	362 (96.8)		
No		7 (6.6)	12 (3.2)		
**After getting vaccine, likely to maintain 6ft**	**482**			0.959	
Yes		97 (91.5)	341 (91.7)		
No		9 (8.5)	31 (8.3)		
**After getting vaccine, likely to attend gatherings**	**482**			0.253	
Yes		65 (61.3)	206 (55.1)		
No		41 (38.7)	168 (44.9)		

*p-value < 0.05 denotes statistical significance

Differences in social influences existed by gender, men and women reported that Hmong community leaders (*P* = .029), government officials (*P* = < 0.000), and healthcare providers (*P* = < 0.001) were influencers to masking. Hmong American men were more likely influenced by Hmong community leaders compared to Hmong American women, in contrast Hmong American women were more likely influenced by government officials and healthcare providers than Hmong American men (Table 5). Hmong community leaders (*P* = .022), government officials (*P* = < 0.000), and healthcare providers (*P* = < 0.000) were influencers to social distancing. Hmong American men were more likely to be influenced by Hmong community leaders to social distancing compared to Hmong American women, whereas Hmong American women were more likely to be influenced by government officials and healthcare providers (Table 5). Hmong American men and women reported that Hmong community leaders influenced their decisions on group gathering behaviors. Hmong American men were more likely to be influenced by Hmong community leaders to attend group gatherings than women (*P* = .026). Social influencers to vaccination by gender showed that Hmong community leaders were social influencers to vaccination, Hmong American men were more likely to be influenced by Hmong community leaders to vaccinate compared to Hmong American women (*P* = .037) (Table 5).

**Table 5:** Social Influences on COVID-19 Mitigation Behaviors by Gender

		Total N	Male (n = 106)	Female (n = 376)	Fisher’s Exact
**Social Influences on Masking**		**482**			
Hmong Community Leader			12 (11.3)	20 (5.3)	0.029*
Family			64 (60.4)	245 (65.2)	0.214
Shaman			5 (4.7)	11 (3)	0.262
Religious leader			7 (6.6)	20 (5.3)	0.38
Government Official			51 (48.1)	250 (66.5)	0.000*
Healthcare Provider			74 (69.8)	317 (84.3)	0.001*
**Social Influences on Social Distancing**		**482**			
Hmong Community Leader			12 (11.3)	19 (5.1)	0.022*
Family			60 (56.6)	220 (58.5)	0.404
Shaman			5 (4.7)	11 (2.9)	0.262
Religious leader			6 (5.7)	16 (4.3)	0.349
Government Official			49 (46.2)	257 (68.4)	0.000*
Healthcare Provider			71 (67)	313 (82.2)	0.000*
**Social influences on Group Gatherings**		**482**			
Hmong Community Leader			22 (20.8)	47 (12.5)	0.026*
Family			64 (60.4)	233 (62)	0.425
Shaman			6 (5.7)	10 (2.7)	0.115
Religious leader			5 (4.7)	13 (3.5)	0.36
Government Official			4 (3.8)	29 (7.7)	0.111
Healthcare Provider			7 (6.6)	32 (8.5)	0.342
**Social Influences on Vaccinations**		**482**			
Hmong Community Leader			12 (11.3)	21 (5.6)	0.037*
Family			63 (59.4)	212 (56.4)	0.327
Shaman			6 (5.7)	8 (2.1)	0.063
Religious leader			3 (2.8)	13 (3.5)	0.518
Government Official			53 (50)	210 (55.9)	0.169
Healthcare Provider			69 (65.1)	267 (71)	0.147

*p-value < 0.05 denotes statistical significance

### Generational status

Masking by generational status showed that there were no differences in masking behaviors reported by first, second, or third generation individuals (Table 6). Generation status and social distancing behaviors showed no differences in social distancing behaviors by generation. For group gathering behaviors in the last 30 days there were differences between first, second and third generation individuals. Third generation individuals were more likely to avoid gatherings of 10 or more in the last 30 days compared to first- and second-generation individuals (*P* = .010). Whereas first generation individuals were more likely to avoid public spaces, gatherings or crowds in the last 30 days compared to second and third generation individuals (*P* = .003). There were no differences between generational status and willingness to avoid family and social gatherings. There were few differences between generational status and vaccination behaviors (Table 6).

**Table 6:** COVID-19 Mitigation Behaviors by Generation Status

		Total N	First Generation (n = 195)	Second Generation (n = 299)	Third Generation (n = 12)	Fisher’s Exact
**Willing to Mask the whole time at gatherings**		**506**				0.341
Yes			164 (84.5)	248 (83.9)	12 (100)	
No			30 (15.5)	51 (17.1)	0 (0)	
**Masking all the time when leaving home**		**506**				0.378
Yes			176 (90.3)	261 (87.6)	12 (100)	
No			19 (9.7)	37 (12.4)	0 (0)	
**Last 30 days worn a mask**		**506**				0.257
Yes			195 (100)	293 (98.3)	12 (100)	
No			0 (0)	5 (1.7)	0 (0)	
**Willing to stay 6ft from people outside the household**		**506**				0.549
Yes			177 (90.7)	265 (88.9)	12 (100)	
No			18 (9.3)	33 (11.1)	0 (0)	
**Maintaining 6ft all the time when leaving home**		**506**				0.051
Yes			120 (61.9)	152 (50.8)	7 (58.3)	
No			74 (38.1)	147 (49.2)	5 (41.7)	
**Last 30 days stay 6ft from people outside the household**		**506**				0.22
Yes			177 (91.2)	258 (86.3)	11 (91.7)	
No			17 (8.8)	41 (13.7)	1 (8.3)	
**Willing to Avoid Family Gatherings**		**506**				0.956
Yes			147 (75.4)	229 (76.6)	9 (75)	
No			48 (24.6)	70 (23.4)	3 (25)	
**Willing to Avoid Social Gatherings**		**506**				0.629
Yes			166 (85.1)	245 (82.2)	11 (91.7)	
No			29 (14.9)	53 (17.8)	1 (8.3)	
**Last 30 days avoided gatherings of 10 or more**		**506**				0.010*
Yes			146 (75.3)	189 (63.2)	10 (83.3)	
No			48 (24.7)	110 (36.8)	2 (16.7)	
**Last 30 days avoided public spaces, gatherings or crowds**		**506**				0.003*
Yes			157 (80.5)	200 (66.9)	9 (75)	
No			38 (19.5)	99 (33.1)	3 (25)	
**Will vaccinate, when COVID-19 vaccine is available**		**506**				0.053
Yes			147 (75.4)	197 (65.9)	10 (83.3)	
No			49 (24.6)	102 (34.1)	2 (16.7)	
**Received the COVID-19 vaccine**		**506**				0.067
Yes			146 (74.9)	195 (65.2)	9 (75)	
No			49 (25.1)	104 (34.8)	3 (25)	
**After getting vaccine, likely to mask**		**506**				0.092
Yes			191 (98.5)	282 (94.6)	12 (100)	
No			3 (1.5)	16 (5.4)	0 (0)	
**After getting vaccine, likely to maintain 6ft**		**506**				0.069
Yes			182 (94.8)	266 (89.3)	11 (91.7)	
No			10 (5.2)	32 (10.7)	1 (8.3)	
**After getting vaccine, likely to attend gatherings**		**506**				0.242
Yes			107 (55.1)	172 (57.7)	4 (33.3)	
No			87 (44.9)	126 (42.3)	8 (66.7)	

*p-value < 0.05 denotes statistical significance

Social influences on masking by generation showed that there was a difference between generation status and government officials as influencers to masking (*P* = < 0.000). First generation individuals were more likely influenced by government officials compared to second and third generation (Table 7). Social influences on social distancing by generation status showed that family were more likely to influence second generation individuals to social distance, compared to first and third generation individuals (*P* = .038). Social influences by generation status show that family were social influencers to group gatherings (Table 7). Third generation individuals were more likely to be influenced by family to attend large group gatherings compared to first-and-second generation individuals (*P* = .041). Social influences on vaccination by generation status show that family were social influencers to vaccination. Third generation individuals were more likely to be influenced by family to vaccinate compared to first-and-second generation individuals (*P* = .011) (Table 7).

**Table 7:** Social Influences on COVID-19 Mitigation Behaviors by Generation Status

	Total N	First Generation (n = 195)	Second Generation (n = 299)	Third Generation (n = 12)	Fisher’s Exact
**Social Influences on Masking**	**506**				
Hmong Community Leader		14 (7.2)	19 (6.6)	1 (8.3)	0.663
Family		117 (60)	205 (68.6)	7 (58.3)	0.119
Shaman		4 (2.1)	12 (4)	1 (8.3)	0.194
Religious leader		8 (4.1)	20 (6.7)	0 (0)	0.41
Government Official		127 (65.1)	187 (62.5)	1 (8.3)	0.000*
Healthcare Provider		152 (77.9)	245 (81.9)	12 (100)	0.127
**Social Influences on Social Distancing**	**506**				
Hmong Community Leader		13 (6.67)	19 (6.4)	1 (8.3)	0.815
Family		101 (51.8)	189 (63.2)	7 (58.3)	0.038*
Shaman		5 (2.6)	11 (3.7)	1 (8.3)	0.314
Religious leader		7 (3.6)	16 (5.4)	0 (0)	0.655
Government Official		126 (64.6)	191 (63.9)	4 (33.3)	0.102
Healthcare Provider		152 (78)	240 (80.3)	11 (91.7)	0.563
**Social influences on Group Gatherings**	**506**				
Hmong Community Leader		28 (14.4)	41 (13.7)	1 (8.3)	0.966
Family		109 (55.9)	194 (64.9)	10 (83.3)	0.041*
Shaman		8 (4.1)	9 (3)	0 (0)	0.746
Religious leader		8 (4.1)	11 (3.7)	0 (0)	0.884
Government Official		17 (8.7)	17 (5.7)	0 (0)	0.3553
Healthcare Provider		21 (10.8)	19 (6.4)	0 (0)	0.17
**Social Influences on Vaccinations**	**506**				
Hmong Community Leader		14 (7.2)	20 (6.7)	2 (16.7)	0.33
Family		95 (48.7)	185 (61.9)	8 (66.7)	0.011*
Shaman		3 (1.5)	11 (3.7)	1 (8.3)	0.154
Religious leader		6 (3.1)	11 (3.7)	0 (0)	0.871
Government Official		110 (59.4)	163 (54.5)	3 (25)	0.106
Healthcare Provider		133 (68.2)	210 (59.8)	8 (66.7)	0.836

*p-value < 0.05 denotes statistical significance

## Discussion

To our knowledge, this is the first investigation of Hmong American mitigation behaviors during the COVID-19 pandemic. We conducted this study when masking mandates were being lifted in various states. Masking mandates at the time of this study had various recommendations, as some states and counties were lifting masking mandates because of the availability of vaccines. COVID-19 vaccination rollout, availability, and requirements included criteria such as age and underlying medical conditions. Individuals not meeting the stated criteria at the time had to wait their turn to get the vaccine. Vaccine availability varied at the state and county level. Our goal was to provide insight on COVID-19 mitigation behaviors of Hmong Americans. We investigated gender, generation status, and sociocultural differences on mitigation behaviors, such as masking, social distancing, group gatherings, and vaccination uptake to describe Hmong American behaviors.

In our study, our participants were among a sample of highly educated, employed, insured, young, mostly healthy, majority second or third generation Hmong Americans that were above the poverty line, with most participants having not been diagnosed with COVID-19 previously. Descriptive statistics showed that our participants were knowledgeable about COVID-19 and participated in the recommended mitigation interventions for COVID-19 such as masking, social distancing, avoiding group gatherings, and getting the COVID-19 vaccine, suggesting possible ceiling effects. COVID-19 mitigation behaviors in our study were shown to be much higher compared to other studies on health prevention behaviors in Hmong Americans [30–33]. Knowledge on COVID-19 in this sample of study participants was relatively higher compared to other studies assessing knowledge on illnesses such as cancer, diabetes, and hypertension [30, 31, 34, 35]. Awareness of the COVID-19 vaccination is relatively higher for this sample compared to other diseases requiring vaccinations. This is likely due to the urgency and need to confront the spread of COVID-19. Vaccination uptake in this study sample were also much higher compared to other studies with vaccination uptake in Hmong Americans [30, 34, 36, 37]. A possible explanation for the high vaccination rate could be because participant characteristics in our study is very different than other samples from other studies. Our sample is younger with higher socioeconomic status and may be required to be vaccinated for school or employment, although it could also be possible that our use of a web survey in English, yielded a relatively higher educated sample compared to the general Hmong American community.

Behaviors by gender and generation status are important as sociocultural norms, expectations, and influences may facilitate or impede mitigation behaviors. Gender roles and expectations in Hmong Americans could have contributed to the differences between men and women as hierarchical and patriarchy practices continue to be prevalent. Attending family and social gatherings are expectations of Hmong men, as their roles may be involved in performing rituals of ancestor worship, weddings, christenings, and family feasts [38, 39]. In our study, we found our participants’ willingness to avoid family and social gatherings varied by gender with men being less willing to avoid than women.

Furthermore, various social influences exist that may affect mitigation attitudes, beliefs, and behaviors. “Social influence is the process where an individual’s attitude, belief, or behaviors are modified from the presence or action of another person, entity, or organization [40].” Social influences had a significant effect on mitigation behaviors in our study. We found that Hmong community leaders were influencers for men with regards to masking, social distancing, group gatherings, and vaccination uptake. Meanwhile, for Hmong American women, healthcare providers were social influencers with masking, and healthcare providers and government officials being the social influencers for social distancing behaviors. These findings could be related to the gender role expectations and sociocultural norms for Hmong Americans. Modeling best practices for public health interventions should include relevance, community participation, stakeholder collaboration, ethical soundness, replicability effectiveness, efficiency, and sustainability [41]. For instance, community participation and stakeholder collaboration in Hmong Americans are especially important when implementing new public health interventions as their influence can highly impact behavior choices in this population and improve their chances of understanding, acceptance, adoption, and adherence. Implementation of public health or health interventions should highlight the importance of these individuals in the Hmong American community and should consult them with future interventions.

Family members were also found to be the strongest proponents of masking, social distancing, group gatherings, and vaccination uptake. During the pandemic, families were the most available source sited of social interaction, connections, and support towards maintaining protective measures against COVID-19. Support from family could play a crucial role in coping mechanisms and norms during the pandemic and may directly influence mitigation behaviors and coping strategies [42]. These findings have several implications for both assessing and implementation of culturally appropriate health messaging, future public health interventions, policy development, and ongoing research with this population.

As we assessed generational status, we observed that different types of social influences on mitigation behaviors varied. The variation in the types of influences could be due to the individual’s acculturative process. Acculturation is a change process that results from constant interaction between two distinct cultures resulting in learned values, behaviors, lifestyles, and language of the host culture [43]. This could explain why there is such differences between, first, second, and third generation mitigation behaviors and the types of influences on those behaviors. As expected, third generation individuals would be influenced by their family, as their worldview may still be limited. Compared to first- and second-generation individuals, social influences may differ based on their life experiences, education and access to information and resources. Therefore, with the ongoing changes to mitigation recommendations in the wake of different variants of COVID-19, additional studies are needed to evaluate the social influences that impact mitigation behaviors to enhance behavior compliance.

Having a better understanding of mitigation behaviors is relevant to ongoing efforts to prevent the spread of a contagious disease such as COVID-19. The COVID-19 virus is continuously evolving, and its impact varies from one group to another. It is still unknown what the long-term effects COVID-19 will have on different communities. Understanding individual comprehension and knowledge on virus transmission and mitigation interventions are crucial to public health research and policy interventions. Hmong Americans’ complex issues with health disparities and engagement in preventative health practices require a full understanding of the contextual and personal characteristics regardless of age, gender, generation, or socioeconomic status. As seen with our study, social influences from family and cultural practices continue to influence health behaviors and attitudes; therefore, culturally acceptable interventions with the use of social influencers are needed to improve efforts to reduce disparities in the Hmong American community.

### Limitations

This study also had some limitations. First, the cross-sectional design of the current study may not provide information about the cause-and-effect relationship. Second, not every Hmong American has access to a computer, internet, has a social media account or know how to use a computer. Therefore, this study is not representative of all Hmong Americans due to the use of a web-based survey and the snowball sampling method used with the help of recruitment partners. Older or elderly Hmong Americans may not know how to navigate a web-based survey and therefore it will limit their ability to participate. Additionally, the survey was only available in English and not representative of non-English speaking Hmong Americans who may be at higher risk for COVID-19. We choose to collect this survey only in English because of the lack of vocabulary and terms in the Hmong language thus limiting the researcher’s ability to accurately interpret the survey items, which could have potentially led to inaccuracies or missing data. Another limitation, we were only able to reach a relatively young population of Hmong American adults through a web survey and future research is needed to reach older Hmong American adults through other methods.

## Conclusion

The COVID-19 pandemic is continuously evolving, and its impact varies from one group to another. It is still unknown what the long-term effects of COVID-19 will have on different communities; however, the importance of understanding how individuals comprehend the virus transmission and mitigation interventions is crucial to public health research and policy interventions. Studies to understand COVID-19 responses on different race and ethnic subgroups are generally limited in the literature. This study among Hmong Americans highlighted potential factors needed to understand the COVID-19 prevention needs of this small community. While cases of COVID-19 continue to flourish in the wake of new coronavirus variants, ongoing education, and health messaging on protective measures and vaccination uptake is needed to keep Hmong Americans safe. The significance of the study findings is that sociocultural context impacts mitigation behaviors. Therefore, our research results suggest an urgent need to improve educational and interventional messages to include the use of Hmong community stakeholders and leaders to enhance protective measures and vaccination uptake. In particular, family-based interventions and messaging that targets the different genders and age groups are needed to help with the acceptance, adoption, and adherence of mitigation behaviors. Insights from this study of Hmong Americans can be used to help inform policies, public health interventions, clinical care, and education interventions, so that it can better serve vulnerable populations experiencing disparities and cultural differences.

## Data Availability

The datasets used and/or analyzed during the current study are available from the corresponding author on reasonable request.
